# A study of the roles of some immunological biomarkers in the diagnosis of rheumatoid arthritis

**DOI:** 10.25122/jml-2023-0158

**Published:** 2023-08

**Authors:** Hasan Abd Ali Khudhair

**Affiliations:** 1 Al-Nasiriyah Technical Institute, Southern Technical University, Ministry of Higher Education and Scientific Research, Thi-Qar, Iraq

**Keywords:** RA, RF, anti-CCP-Abs, anti-MCV-Abs, APF, AKA, Abs: Antibodies, ACPAs: Anti-citrullinated Protein Antibodies, ACR: American College of Rheumatology, Ag: Antigen, AKA: Anti-keratin Antibodies, APF: Anti-perinuclear Factor, AS: Ankylosing Spondylitis, CCP: Cyclic Citrullinated Peptide, ELISA: Enzyme-Linked Immunosorbent Assay, EULAR: European League Against Rheumatism, HC: Healthy Control, HLA: Human Leukocyte Antigen, IgG: Immunoglobulin Gamma, MCV: Mutated Citrullinated Vimentin, ml: Milliliter, n: Number, NPV: Negative Predictive Values, PC: Positive Control, PPV: Positive Predictive Values, RA: Rheumatoid Arthritis, RF: Rheumatoid Factors, SEs: Shared Epitopes, SLE: Systemic Lupus Erythematosus, SS: Sjögren's Syndrome, U: Unit

## Abstract

Autoimmune rheumatoid arthritis (RA) is a systemic condition closely correlated with a variety of autoantibodies (Abs) that could be considered diagnostic and prognostic markers. The current research was designed to detect the diagnostic values for a number (n) of these auto-Abs in RA detection and to evaluate the accuracy of a combined diagnostic scheme. This prospective study was conducted between September 2021 and August 2022 and included 110 subjects with RA, 70 individuals with other autoimmune disorders as positive controls (PC), and 50 unrelated, apparently healthy individuals as healthy controls (HC). The eligibility criteria for all study groups were followed stringently. An enzyme-linked immunosorbent assay (ELISA) was employed to measure rheumatoid factors (RF), cyclic citrullinated peptide antibodies (CCP-Abs), mutated citrullinated vimentin antibodies (MCV-Abs), anti-perinuclear factor antibodies (APF-Abs), and anti-keratin antibodies (AKA). We calculated the specificity, sensitivity, and predictive values of all auto-Abs. Significantly higher levels of anti-CCP-Abs, anti-MCV-Abs, APF-Abs, and AKAs were reported in the RA patients compared to the HC and PC subjects. RF levels, however, were only statistically elevated when compared to the HC individuals. Anti-APF-Abs had a higher sensitivity rate (70.9%), and anti-CCP-Abs had a higher specificity rate (94.16%) compared to other auto-Abs, whereas the combined detection scheme revealed a higher sensitivity (81.81%) and excellent specificity (90.83%) compared to the two former auto-Abs. Anti-perinuclear factor-Ab was a highly sensitive test, and CCP-Ab was a surpassingly specific assay for identifying RA. Furthermore, the combined detection scheme is an essential serological approach for RA diagnosis and crucial in differentiating this disease from other autoimmune diseases, thus promoting early diagnosis and treatment.

## INTRODUCTION

Rheumatoid arthritis is an immune-reactive condition that principally involves the joints, with an estimated worldwide prevalence of 1% [1]. Possible risk factors include gender, age, inheritance, and exposure to pollutants (tobacco, pollution, and industrial risks) [2]. Rheumatoid arthritis is triggered by a complicated interaction between immunological, genotypic, ecological, and other factors. Despite the role of these factors, the precise cause of RA is yet controversial, and the prognosis is guarded [3]. The initial manifestations of RA can range from joint aches and swelling in the proximal interphalangeal and metacarpophalangeal joints to progressive involvement of major joints, such as the elbow, ankle, and knee. Ultimately, high levels of active white blood cells within the membrane of the synovial fluid result in inflammation and hyperplasia, which may result in the gradual deterioration of bone and cartilage [4]. If left without treatment, it may result in a variety of adverse effects, including irreversible damage of the joints that need an arthroplasty, Felty syndrome, which needs a splenectomy, and rheumatic vasculitis. Since there is no curative treatment for RA, therapy aims were to decrease inflammation and delay further complications [2]. Clinical observations and extra diagnostic techniques are used for RA diagnosis. A diagnosis cannot be confirmed with imaging, a single laboratory, or a histological test alone. Because the disease course is exceptionally varied and unpredictable, highly sensitive and specific diagnostic assays are required. Previously published data have shown that treatment interventions aimed at effectively controlling the disease during the early stages of RA lead to reduced joint deterioration and more favorable prognostic outcomes [5]. However, diagnosis may be challenging in the earliest phases when clinical and radiological evidence may not be prominent. Therefore, a sensitive and specific immunological assay could be valuable in differentiating RA from other rheumatic diseases. The current study was carried out to identify more accurate prognostic and diagnostic biomarkers for early RA. As RA is an immune inflammatory disease, numerous auto-Abs develop in the patient’s serum as the disease progresses [6]. Serological diagnosis typically relies on the presence of RF auto-Abs that recognize the Fc part of immunoglobulin gamma (IgG) molecules as their antigen (Ag). Nevertheless, RF is not restricted to this disease and can additionally present in other arthritic conditions, transmissible conditions, and normal people [7].

Anti-citrullinated protein-Abs (ACPAs) were recognized as highly specific immunological biomarkers for RA and are preferred over the RF assay in RA diagnosis [7, 8]. This auto-Abs group consists of an overlapped array of auto-Abs that depend on arginine residue citrullination. It is composed of APF-Abs, AKA, anti-filaggrin-Abs, anti-Sa, and anti-CCP-Abs [5]. Each auto-Abs Ag is structurally identical with citrullinated molecules arising from arginine alterations post-translationally and are therefore referred to as ACPAs, especially prevalent in patients with RA [9]. Nevertheless, only anti-CCP-Abs are used in clinical practices [5]. The Abs against CCP that target filaggrin-citrulline system Ags are highly specific compared to RF for detecting RA, but the sensitivity of this assay is similar to RF. Studies indicate that anti-CCP auto-Abs are a better diagnostic tool than RF, with good positive and negative predictive values for RA identification. The incidence of anti-CCP-Abs correlates with disease severity and predicts radiographic joint destruction in the initial RA [7, 10]. Citrullinated vimentin, the target Ag for anti-Sa Abs, has been identified as a novel member of the ACPAs family [5]. Vimentin is an intermediate thread that is abundantly present in the synovium. It is produced and citrullinated by apoptotic monocytes and is prominent in the RA synovial milieu due to poor elimination. Therefore, citrullinated vimentin is being recognized as a potential auto-Ag with diagnostic ability, and an ELISA-detection auto-Abs specific for recombinant MCV was developed. Auto-Abs against MCV are being postulated to have considerable adjunctive diagnostics utility in acute and chronic RA. Nevertheless, a definite conclusion on the practical and diagnostics values of anti-MCV-Abs in RA compared with other ACPAs remain unavailable, and the relatively small specimen numbers of individuals recruited in most published trials limit findings and the data evaluation [11, 12].

Auto-Abs against perinuclear factor react with Ags of granules of cytoplasm on human oral mucosal tissues. Due to moderate sensitivity and high specificity, APF auto-Abs are considered a crucial serum test for the confirmation of RA diagnosis and an effective immunological biomarker that differentiates this form of arthritis from other autoimmune rheumatic disorders. Anti-perinuclear factor auto-Abs have also been detected in seronegative RA subjects. This auto-Ab does not vary by gender, age, or disease duration and arises early or maybe preceding the first episode of clinical RA. Anti-perinuclear factor has a similar percentage of sensitivity as RF but has greater specificity than RF in confirming the presence of RA. As APF can be found in rheumatic nodules and joint fluid and has been associated with the incidence of RF, it is useful in the detection of acute RA and may be regarded as the main criterion for RA classification [13]. Therefore, the current study investigated APF as a biomarker for RA diagnosis. Anti-keratin auto-Abs that target citrullinated proteins had a specificity range of 79%–100% as a biomarker for detecting arthritis and a range of sensitivity between 20% and 80% [14]. As a result, the present study was performed to determine if AKA could serve as an adjunct diagnostic blood biomarker for RA. Despite the presence of the various auto-Abs mentioned above, there is still a need for a serological marker and/or test with higher sensitivity and specificity to achieve early diagnosis and implement intensive treatment for RA, thereby enabling effective disease control. When considering each of the individual RA-associated auto-Abs alone, they exhibit poor sensitivity and limited specificity. However, combining the detection of these auto-Abs could enhance their diagnostic value significantly. Therefore, the present research aimed to assess the diagnostic values of several immunological biomarkers (RF-Abs, CCP-Abs, MCV-Abs, APF-Abs, and AKA) in confirming the presence of RA and to determine the diagnostic accuracy of the combination detection scheme among them.

## MATERIALS AND METHODS

### Participants/study design

A prospective study was executed between September 2021 and August 2022, including 110 patients with RA (99 women and 11 men) within 23-70 years of age, meeting the categorization criteria of the European League Against Rheumatism (EULAR) and the 2010 American College of Rheumatology (ACR) [15], considered the reference standard. Accordingly, the EULAR and the ACR introduced updated categorization norms in 2010, which emphasize RA features that manifest at the onset of arthritis, such as ACPAs, a marker that indicates severe disorders. On the contrary, the 1987 American Rheumatism Association criterion differentiated confirmed RA subjects from individuals with other types of rheumatism and distinguished those with advanced disease [15].

The control group (PC group) included 70 individuals with clinical manifestations of other autoimmune diseases, including Sjögren's syndrome (SS) (n=20), ankylosing spondylitis (AS) (n=20), and systemic lupus erythematosus (SLE) (n=30). The 2002 American-European Consensus Group revised criterion was used to identify SS patients [16], subjects with AS were classified based on the 1984 New York revised criterion [17], whereas SLE patients were identified based on the 1997 ACR revised criterion [18]. All participants were selected from the Clinic of Rheumatology, Teaching Hospital of Al-Hussein, Al-Nasiriyah, Iraq. Patients with one or more of the following criteria were omitted from the present research: under non-steroidal anti-inflammatory agents in the previous seven days, use of corticosteroid medications in the previous four weeks, use of biological agents recently or before this, current transfusions of blood and its products within the preceding six months, recently performed surgery within the last six months and the existence of any other autoimmune or chronic diseases.

Moreover, 50 unrelated, apparently healthy volunteers were randomly selected as the HC group, with gender and age matching the RA subjects. The inclusion criteria for the HC group were as follows: none of the above-mentioned patient exclusion criteria, no history of autoimmune or chronic diseases, and no history of any conditions associated with RA. Additionally, individuals with relatively mild infections were also excluded from the HC group.

### Sample collection

Peripheral venous blood (3 to 4 milliliters (ml)) was obtained from all participants using vein piercing. Collected specimens were allowed to coagulate at room temperature and then centrifuged at 1500 xg for ten minutes to obtain sera, which were stored at -80 degrees Celsius until further serological investigations.

### Immunological tests

The serological tests were conducted according to the recommendations provided in the kits' manufacturer manuals by two highly skilled laboratory technicians at the Public Health Laboratory in Al-Nasiriyah City. The laboratory technicians conducting the assays were blinded to the results of other tests and had no access to information about the clinical manifestations of the subjects. The serological tests included the detection of the following auto-anti-Abs: RF-Abs (IgG), anti-CCP (IgG) Abs, anti-MCV (IgG) Abs, APF (IgG) Abs, and AKA (IgG) Abs.

For the detection of RF-Abs (IgG), a specific ELISA kit (Demeditec, Germany, Reference: DE7640) was used, and the results were quantified in Units (U)/ml. A concentration equal to or higher than 20 U/ml was regarded as positive for RF-Abs.

Anti-CCP (IgG) and anti-MCV (IgG) Abs were detected using human ELISA kits from Cusabio, China, with catalog numbers CSB-E09077h and CSB-E09565h, respectively. The cut-off values for anti-CCP and anti-MCV Abs were set at ≥2.1, and results equal to or greater than this value were considered positive.

To detect APF (IgG) Abs and AKA (IgG) Abs, ELISA kits from MyBioSource, United States of America, with catalog numbers MBS161433 and MBS727108, respectively, were used. The concentrations of these biomarkers were measured in nanogram/ml, and the cut-off values for APF and AKA Abs were determined with a 95% confidence interval using the serum samples from the HC group mentioned earlier.

Using standardized ELISA kits and applying specific cut-off values ensured consistent and reliable results in detecting the various auto-Abs, which were essential for accurately assessing their diagnostic values in the study of RA.

### Combination scheme

The five auto-Abs were evaluated individually and combined arbitrarily. For the combination form, the results were considered positive for RA if three or more of the five auto-Abs showed positive values. Conversely, the results were considered negative if three or more of the five auto-Abs showed negative values.

### Statistical methods

Statistical analysis was performed using the Statistical Package for Social Sciences (SPSS) version 25. The chi-square test was employed to assess the frequency distributions of the parameters between different research categories. Additionally, Receiver Operating Characteristic (ROC) curves were performed to calculate the degrees of specificity, sensitivity, and predictive values for all the research biomarkers. The statistical significance was determined by considering a p-value lower than 0.05 as statistically valuable.

## RESULTS

The current investigation was conducted between September 2021 and August 2022, involving a total of 110 patients with clinical manifestations of RA, including 99 women and 11 men, with ages ranging from 23 to 70 years. Additionally, there were 70 individuals with signs and symptoms of other autoimmune disorders, including 30 with SLE, 20 with SS, and 20 with AS. Furthermore, 50 apparently healthy unrelated individuals with gender and age matching the RA patients were included in the HC group.

The findings of the research revealed a significant (p<0.05) elevation in the frequency of RF positivity in most RA subjects (66.4%) and patients with other autoimmune disorders (PC group) (58.6%) compared to a lower positivity rate in the HC group (8%). However, no statistically significant difference (p>0.05) was reported between the RA and PC groups for this biomarker.

Regarding serum anti-CCP Abs, a higher percentage of RA patients (69.1%) showed positive results, which were significantly different from the PC patients (8.6%) and HC group (2%). There was no statistically significant difference in anti-CCP Ab positivity between the PC and HC groups (p>0.05). Most RA subjects (67.3%) had serum anti-MCV-Abs, which was significantly higher than the PC subjects (17.1%) and HC subjects (6%) (p<0.05). There was no statistically significant difference in anti-MCV Ab positivity between the PC and HC groups (p>0.05).

The frequency of positive serum APF-Abs was statistically (p<0.05) higher among RA patients 78/110 (70.9%) compared to the PC 12/70 (17.1%) and HC 1/50 (2%) groups. However, the difference in APF Ab positivity between the PC and HC groups was not statistically significant (p>0.05). Finally, there was a statistically significant increase in AKA positivity among individuals with RA (47.3%) compared to the PC group (10%) and the HC group (2%). There was no statistically significant difference in AKA positivity between PC and HC individuals (p>0.05) (Table 1).

**Table 1:** Frequency of study biomarkers among research participants

Biomarkers / Groups		RA (n=110)		PC (n=70)		HC (n=50)		Total (n=230)		Statistic (p-value)*
		FR	%	FR	%	FR	%	FR	%	
**RF-Ab**	**Positive**	73	66.4	41	58.6	4	8	118	51.3	**RA x PC: >0.05** **RA, PC x HC: <0.05**
	**Negative**	37	33.6	29	41.4	46	92	112	48.7	
**Anti-CCP-Ab**	**Positive**	76	69.1	6	8.6	1	2	83	36.1	**RA x PC, HC: <0.05** **PC x HC: >0.05**
	**Negative**	34	30.9	64	91.4	49	98	147	63.9	
**Anti-MCV-Ab**	**Positive**	74	67.3	12	17.1	3	6	89	38.7	**RA x PC, HC: <0.05** **PC x HC: >0.05**
	**Negative**	36	32.7	58	82.9	47	94	141	61.3	
**APF-Ab**	**Positive**	78	70.9	12	17.1	1	2	91	39.6	**RA x PC, HC: <0.05** **PC x HC: >0.05**
	**Negative**	32	29.1	58	82.9	49	98	139	60.4	
**AKA**	**Positive**	52	47.3	7	10	1	2	60	26.1	**RA x PC, HC: <0.05** **PC x HC: >0.05**
	**Negative**	58	52.7	63	90	49	98	170	73.9	

RA: rheumatoid arthritis, PC: positive control, HC: healthy control, n: numbers, FR: frequencies, %: percentage, RF: rheumatoid factors, Ab: antibody, AKA: anti-keratin antibody, MCV: mutated citrullinated vimentin, APF: anti-perinuclear factor, CCP: cyclic citrullinated peptide, and x: indicated the comparison.

* Interpretation: p<0.05 indicates the differences are statistically significant, p ≥ 0.05 indicates the differences are not statistically significant.

**Figure 1:**
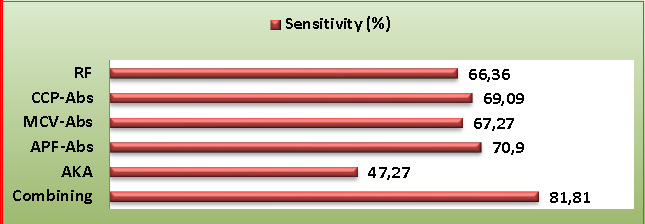
Sensitivity of study biomarkers and combining scheme for rheumatoid arthritis diagnosis (%: percent, RF: rheumatoid factors, Abs: antibody, MCV: mutated citrullinated vimentin, AKA: anti-keratin Ab, APF: anti-perinuclear factor, and CCP: cyclic citrullinated peptide).

In reference to the 2010 ACR and the EULAR diagnostic criteria for RA (as the reference test) [15], the specificity, positive predictive values (PPV), sensitivity, and negative predictive values (NPV) of all investigated biomarkers were calculated, as well as the combining scheme. The highest sensitivity was recorded with combining scheme and APF-Abs (81.81% and 70.9%, respectively), followed by anti-CCP-Abs, MCV-Abs, and RF (69.09%, 67.27%, and 66.36%, respectively). The lowest sensitivity was recorded with AKA (47.27%) (Figure 1).

**Figure 2:**
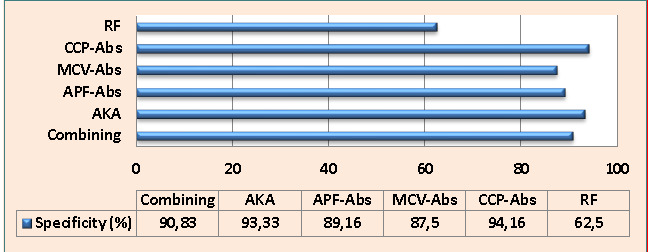
Specificity of study biomarkers and combining scheme for rheumatoid arthritis diagnosis (CCP: cyclic citrullinated peptide, %: percent, RF: rheumatoid factors, APF: anti-perinuclear factor, Abs: antibody, AKA: anti-keratin antibody, and MCV: mutated citrullinated vimentin).

CCP-Abs, AKA, and combining scheme had the highest specificity rate (94.16%, 93.33%, and 90.83%, respectively), followed by APF-Abs and MCV-Abs (89.16% and 87.5%, respectively), whereas RF had the lowest specificity rate (62.5%) (Figure 2).

**Figure 3:**
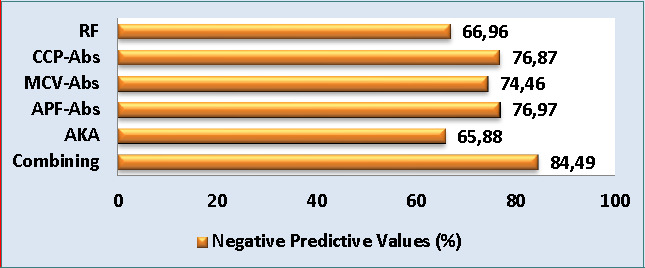
The negative predictive values of all study biomarkers and combining scheme for rheumatoid arthritis diagnosis (%: percent, CCP: cyclic citrullinated peptide, RF: rheumatoid factors, APF: anti-perinuclear factor, Abs: antibody, AKA: anti-keratin antibody, and MCV: mutated citrullinated vimentin).

The current study found that the combining scheme had the highest NPV at 84.49%, followed by APF-Abs, anti-CCP-Abs, and MCV-Abs with NPVs of 76.97%, 76.87%, and 74.46%, respectively. On the other hand, the NPV of RF and AKA were 66.96% and 65.88%, respectively (Figure 3).

**Figure 4:**
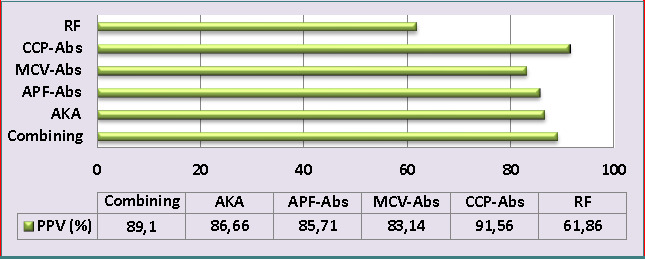
The positive predictive values of all study biomarkers and combining scheme for rheumatoid arthritis diagnosis (PPV: positive predictive values, MCV: mutated citrullinated vimentin, %: percent, RF: rheumatoid factors, APF: anti-perinuclear factor, Abs: antibody, AKA: anti-keratin antibody, and CCP: cyclic citrullinated peptide).

Concerning PPV, the findings revealed (Figure 4) a higher PPV for anti-CCP-Abs at 91.56%, followed by the combining scheme at 89.1%, AKA at 86.66%, APF-Abs at 85.71%, and MCV-Abs at 83.14%. The lowest PPV was reported for the RF biomarker at 61.86%.

## DISCUSSION

Autoimmune RA is a widespread, longstanding reactive joint disorder affecting between 0.5% and 1% of the global population. Evidence strongly suggests that early interventions in RA can significantly improve disease management, reduce joint deterioration, and improve long-term outcomes. Consequently, there is a pressing need for a diagnostic test with high accuracy to detect RA in its early stages. The primary auto-Abs related to RA is RF, among the most reliable and commonly employed RA serum assay. Nevertheless, it is not accurate for detecting RA at the preclinical stage since RF is found in normal old people or individuals with other immunological and infectious conditions [19].

Consistent with these findings, our current investigation revealed that RF was predominantly positive in individuals with RA (66.4%) and PC (58.6%), compared to HC (8%). This outcome was in line with the findings of Paknikar *et al*. [20], who reported a 53% RF positivity rate among individuals with RA. Cyclic citrullinated peptide-Abs offer a major boost for precise diagnosis of RA in individuals with inflammation-related arthritis before disease progression. It was statistically correlated with various indices of RA severity and activity [10, 21]. In line with these findings, our study demonstrated a statistically significant elevation in the positive frequency of anti-CCP-Abs among RA patients compared to PC and HC groups (Table 1). This was consistent with the observations of Osman *et al*. [22], who also recorded elevated levels of MCV-Abs and CCP-Abs in RA subjects compared to osteoarthritis and control groups. Compared to PC and HC individuals, the percentage of anti-MCV-Abs positivity was statistically (p<0.05) elevated in RA subjects (Table 1). Similarly, Nigm *et al*. [23] and several other previous studies have shown a statistical rise in circulating anti-MCV-Abs levels in RA subjects compared to controls [24]. The underlying mechanism could be related to vimentin’s role in initiating the early immunological reaction in RA [24, 25]. It stimulates T lymphocytes by interacting with human leukocyte Ag (HLA)-DR4 on the Ag-presenting cell surface, and it might have a role in the RA pathophysiology [24].

The current study demonstrated a significant increase (p<0.05) in the APF-Abs positive rate among the RA group compared to the PC and HC groups (Table 1). This result is analogous to another published study highlighting the effectiveness of APF-Abs as an immunological test for detecting RA disease and a valuable immunological biomarker for differentiating RA from other arthritis-related diseases [13]. The percentage of AKA was statistically (p<0.05) elevated among RA individuals in comparison with the PC and HC groups (Table 1). Consistent with these findings, Li and Sun [21] recorded that the percentage of AKA positivity was higher among RA subjects than the control subjects. Additionally, Wang *et al*. [14] found that AKA was an effective circulatory diagnostic biomarker for RA.

It is becoming highly essential to identify RA at the earliest stages to start therapy and prevent severe joint tissue damage. Consequently, a sensitive and specific immunological assay is required. Rheumatoid factors are often considered a moderately sensitive (66.36%) and relatively less specific (62.5%) biomarker for RA diagnosis (Figures 1, 2). In parallel to these findings, Liu *et al*. [5] demonstrated that RF had a sensitivity of 72.4%, while Soós *et al*. [26] reported a low specificity for this biomarker. The explanation for these results lies in the fact that RF can additionally be present in subjects with other inflammatory immune-reactive diseases, infectious diseases, and to some extent, in healthy elderly individuals. Regardless of its moderate specificity and sensitivity, the existence of RF is frequently considered a biomarker for RA identification [19].

Cyclic citrullinated peptide Abs play a crucial role in the precise detection of RA in individuals with inflammatory-associated arthritis before it progresses. These Abs have been found to be statistically related to many indices of RA severity and activity. Although anti-CCP-Abs tests are efficient and commonly used for RA diagnosis, their sensitivity is restricted in individuals with an initial phase of RA [2]. In our study, anti-CCP-Abs showed high specificity (94.16%) but relatively lower sensitivity (69.09%) (Figures 1, 2). Despite the moderate sensitivity, anti-CCP-Abs remain a valuable biomarker with a strong capability to identify both erosive and non-erosive RA cases [27]. The excellent specificity of anti-CCP-Abs is undeniable and contributes to its significance in the diagnostic process. Furthermore, these findings were consistent with another report by Liu *et al*. [5], which reported the sensitivity of anti-CCP-auto-Abs of 61.8%. Concerning the utility of MCV-Abs in the detection and prediction of RA, the current findings showed higher diagnostic specificity (87.5%) and moderate sensitivity (67.27%) of MCV-Abs in the detection of RA (Figures 1, 2). These results were consistent with another study [26], which found a sensitivity of 75.6% and specificity of 91.5% for MCV-Abs in RA patients. Similarly, Mohammed *et al*. [28] reported that anti-MCV-Abs had a specificity of 83% and sensitivity of 63%. In another study, the anti-MCV-Abs test showed a good level of sensitivity (74%) and a higher level of specificity (96.1%) [29]. The anti-perinuclear factor was a useful immunological assay for RA diagnosis and a valuable immunological biomarker for differentiating RA from other rheumatoid diseases [13]. In agreement with the above-cited study, serum APF-Abs exhibited a good diagnostic sensitivity (70.9%) and higher diagnostic specificity (89.16%) (Figures 1, 2). Previous research regarding APF-Abs among RA patients reported a specificity of 98.4% and a sensitivity of 43.4% [30]. Similarly, Abedian *et al*. [13] recorded APF-Abs specificity of 94.3% and sensitivity of 71.2%. The results of the current study exhibited a weak sensitivity (47.27%) and excellent specificity (93.33%) of AKA in the diagnosis of RA (Figures 1, 2).

In line with the current findings, a previous meta-analysis reported that AKA had a cumulative specificity of 95% and cumulative sensitivity of 42% within the European group. However, in the Asian population, the specificity was 98%, and the sensitivity was 46% [14]. The current study findings confirmed that AKA was a weak serological biomarker for RA diagnosis due to its low sensitivity percentage. The variations in sensitivity observed between the current study and previous studies may be attributed to several factors, such as different serum dilutions tested, different cut-off values, and differences in the techniques used.

It is essential to recognize that RA is a multifactorial disease, and the generation of ACPAs is influenced by a complex interplay of inherited, ecological, infectious, dietary, lifestyle, and stochastic factors. Human leukocyte Ag-DR, mostly HLA-DR4 and HLA-DR1, identified as shared epitopes (SEs), are the most hereditary predisposition genes consistent with ACPA-positivity in RA subjects. Since SE is an important trigger for the emergence of ACPAs, it is speculated that SE affects the course of RA due to ACPAs onset. Moreover, smoking in the coexistence of the HLA-DR-SE gene could enhance specific immunological responses against citrullinated Ags. In RA, the external milieu serves as a stimulant for the emergence of ACPAs, and epigenetic factors surveillance might connect the ecology with the genetic matter. Auto-Abs reaction against citrullinated Ags is influenced by interactions between genes and the environment. Substantial evidence underscores the role of *Porphyromonas gingivalis, Aggregatibacter actinomycetemcomitans*, and Epstein-Barr virus as immunological triggers of autoimmunity in RA. Finally, the consumption of omega-three fatty acids may not only reduce the chance of ACPAs emergence but can also delay the development of RA even though ACPAs have been identified [31].

This study evaluated the sensitivity, specificity, NPV, and PPV of various auto-Abs in a combined scheme, as mentioned in the methodology section. Overall, the sensitivity of combined detection was improved by 15.45%, 12.72%, 14.54%, 10.91%, and 34.54% compared to individual sensitivity of RF, anti-CCP-Abs, MCV-Abs, APF-Abs, and AKA, respectively, whereas the specificity value remained within excellent percent (90.83%) compared with the specificity of individual auto-Abs (Figures 1, 2). Liu *et al*. [5] reported that the combining of CCP-auto-Abs and MCV-auto-Abs resulted in higher specificity and sensitivity, supporting the idea that combining different auto-Abs can improve diagnostic accuracy. Similarly, Soós *et al*. [26] confirmed that using a combination of MCV-auto-Abs and CCP-auto-Abs assays could enhance the RA laboratory diagnosis. According to Sun *et al*. [4] findings, the following combined tests possess a potent diagnostic utility for the RA diagnosis: MCV-Abs + CCP-Abs, RF + CCP-Abs, and RF + CCP-Abs +immunoglobulin heavy chain binding protein.

## CONCLUSIONS

Anti-perinuclear factor-Ab was a highly sensitive test, and anti-CCP-Ab was a more specific assay for RA detection compared to other serological biomarkers. The combined detection scheme showed a higher sensitivity value compared to using anti-APF-Ab alone. It can be concluded that a combined detection scheme is a valuable diagnostic strategy for RA detection and crucial in differentiating this disease from other autoimmune diseases, thus promoting early diagnosis and management.

## Personal thanks

The author intends to offer his gratitude to the staff of the Rheumatology Clinic, Teaching Hospital of Al-Hussein in Al-Nasiriyah City for their collaboration.
